# A Case of Hooping Cough Superseded by the Small-Pox

**Published:** 1802-11-01

**Authors:** T. V. Okes


### *A Case of Hooping Cough superseded by the Small-pox; communicated by* Mr. T. V. Okes.

A Child, a year and quarter old, at Milton, near Cambridge, had been for ſeveral months afflicted with the Hooping Cough. The cough was more violent and more ſhockingly convulſive than I ever obſerved it to be in any other ſubject. Emetics and medicines uſually preſcribed in ſimilar caſes were duly adminiſtered, and although the returns of the cough were afterwards leſs frequent, the hooping and convulſion connected with it did not yield to any of the means employed.

The mother having been ſeized with the natural ſmall-pox, it was thought adviſable to inoculate the child; and ſome matter for this purpoſe, taken from the mother (three days after the eruption) was inſerted in both the arms of the child. At the end of ſix days the eruptive fever came on, and was ſucceeded by a moderate quantity of puſtules: In ſhort, every ſymptom belonging to the ſmall-pox appeared to be of the moſt favourable kind; but what is more particularly to be remarked upon this occaſion is, *that as soon as the eruptive fever shewed itself, the cough and its concomitant symptoms entirely ceased and never more returned.*

I do not think myſelf enabled to make any important deduction from this ſolitary fact; but the ſudden ceſſation of the cough and the other characteriſtics of the original diſeaſe upon the approach of the eruptive fever, ſeems to be a very ſtriking circumſtance, and may perhaps point out ſomething worthy of conſideration as to the treatment to be adopted in the latter ſtages of the Hooping Cough.

*Sept.* 25, 1802.

